# Follicular Abscess of the Urethra

**Published:** 1880-05

**Authors:** Fessenden N. Otis

**Affiliations:** New York, delivered at the College of Physicians and Surgeons, New York City


### Article II.

Follicular Abscess of the Urethra. A Clinical Lecture, by Prof. Fessenden N. Otis, of New York, delivered at the College of Physicians and Surgeons, New York City.

The patient presented to the class was suffering from gonorrhœa, and besides, called attention to a small hard "lump" situated on the under side of the penis, in the median line and about one inch and a half from the meatus urinarius. In reference to his gonorrhœa, the man stoutly denied recent venereal contact, although he acknowledged that a previous clap which he had three or four years ago, was of venereal origin. In this connection, Prof. Otis said, "It really makes no difference in your treatment of a gonorrhœa whether your patient says he got it from the seat of a water-closet or not, nor are you, as far as treatment is concerned, to always credit the various stories these patients tell about the origin of their trouble. You have the diseased state before you, and you are to treat it as it presents itself. And yet there may be a gonorrhœa without venereal contact, which behaves exactly like a true gonorrhœa, and may have all the complications. In this particular instance, I do not see any special reason for doubting this man's word when he denies venereal contact. I once knew a case of this false gonorrhœa, which happened in the family of a gentleman who had several children. One of his children had a purulent ophthalmia and it was customary for the gentleman to attend to the necessary manipulations about the eyes himself. Another of his children had some trouble with the penis, and this also the gentleman cared for. It was his custom to attend to the eyes first. After a day or two, however, a purulent discharge appeared from the other child's penis, and this was followed by swelled testicle. It was a case of gonorrhœa produced by infection from the ophthalmia. In fact, it is a common thing to have a gonorrhœa develop itself in a man who has had a stricture for ten or fifteen years, without venereal contact. I have seen men marry who had a slight gleet, and who were permitted to marry by their physicians, who communicated a gonorrhœa to the wife eight or ten days after marriage. I knew of one very fatal case of this kind, where a gentleman, in the first week after marriage, infected his wife's eye with gonorrhœa, which eye she lost, and shortly before the end of the honeymoon, the other also. This man may have a gonorrhœa without venereal contact. It is then a gonorrhœa by mediate and not direct contagion. In the mediate, there is a shorter course. It does not take a long time for the poison of a gonorrhœa, when exposed to the air, to be destroyed, which perhaps explains why so few of us become infected by this mediate contagion, and also why these mediate gonorrhœas run a shorter course.

The man has told us of this tumor on the under side of his penis. It is situated an inch and a quarter from the meatus. I hold it under my finger. It is hard, presents no sign of fluctuation, and gives the man no pain. He tells us that it has been aspirated by his physician, but that no pus was found, so that, whatever it is, it is a neoplasm, but has not a purulent center. It is too far forward for Cowper's glands. He says that at first it was about the size of the head of a small pin, and gradually grew larger. There are several things that must not be overlooked, which may have produced this tumor. "Have you ever had any sores on your penis?" "Yes." "Or lump in your groin?" "Yes." "Or eruption on your body?" "No." "Ever had sores in your mouth, or ulcerated throat?" "No, sir." "Hair come out a good deal?" "Yes, sir." The patient does not seem to have had any eye trouble or headache, or signs of nodes. I am trying to connect this tumor with syphilis—an attack which happened a long time ago, not a recent attack. If this growth is of syphilitic character, it is a gummous tumor. Last winter, you will remember, we had such a case, where the tumor ulcerated and was supposed to be an infecting sore. This growth, however, is not a gummy tumor. There is not the characteristic enlargement of the glands nor other corroborative evidence. What comes next, then? The most likely thing that I know about is a follicular inflammation. The urethra, as you know, is studded with follicles. They are very minute—not much larger than a cambric needle. They may become involved in the inflammation of the urethra, and a minute suppuration occur in the mouth of the follicle, which then may be closed—plugged up. This little molecule of matter burrows along and forms a sinus, making an independent opening, the pus being pushed back into the urethra from the outside. This is more likely to occur within an inch or half an inch from the meatus. The urethral end of the follicle becomes sealed up, and you have a follicular sinus left. A case of this kind was reported by me as far back, I think, as 1870. I remember a case of a gentleman with gleet, who had been treated a long time, but without success. On examining him, I found a little white point, the size of a needle point, an inch behind the meatus. It had been there, he said, for a long time. Occasionally matter came out. I introduced a fine probe, and after injecting it with indigo, found the stain of indigo on cotton which I had placed in the urethra previously. I then sharpened a hypodermic needle down to a fine point, and introducing it into this little sinus, injected a forty grain solution of the nitrate of silver, and the gentleman had no more trouble. The sinus healed up, and his gleet got well. This little canal had been the seat of a gonorrhœa all the time, and the ordinary injections never reached the inflammation. I have found in cases where the same thing occurred, abscesses formed, which in some instances were absorbed, but in fifteen or twenty cases the matter came to the surface.

Dittel has shown the gravity of these cases where they occur in the deeper portions of the urethra, where, as a result of perforation, independently of stricture, extravasation of urine has occurred, which has proved fatal. This has been traced back to a follicle in the neighborhood of the membranous urethra, the urine being let through in very small quantity at first, and then in larger quantities.

These bunches, then, come from the urethra, following a gonorrhœa. They are not independent, but are the result of a suppuration. Why does this occur? I believe, and have found in every case, that they always occur in a follicle situated *behind a stricture;* that the condition of things which exists behind a stricture is the condition which invites this inflammation. Where there has existed a long standing irritation, it is not wonderful that there should be inflammatory action excited.

Dittel noticed that in all these cases there were *rings* of mucus in the urine. You have rings of mucus because they come from a circular point of irritation, which holds the mucus in the form of a ring. These rings of mucus are one of the signs of stricture, and these rings come from behind a stricture. So whenever you find a sudden swelling in the vicinity of the urethra, examine for stricture. I never fail to find it, and shall find it in this case before you. My own impression is that here we have a stricture which has occurred after an old gonorrhœa, and after a new irritation it has resulted in a neoplasm, an inflammation set up by the continuous irritation, and this is the result of stricture.

Some years ago I called the attention of the profession to the case of a gentleman from a neighboring State who was suffering from an obscure disease of the genito-urinary apparatus. It was supposed to be malignant, because of the ulceration of penis and scrotum. He had had a swelling in the scrotum, which kept increasing very slowly for five years, until it got to be of the size of a child's head at term, very red and insensitive. He did not seem to be troubled at all by it. However, he had an attack of malarial fever, and was reduced very low, and while in this condition the bottom of the scrotum began to slough, and a nasty, dirty abscess appeared, which seemed to be the result of some malignant process. However, it became healthy looking, and finally healed up, and then another abscess formed an inch above, and in ten days opened just as the first, and followed the same course; then another and another, each one higher up, until finally another abscess formed on the side of the penis, which was followed by such inflammation that the foreskin became restricted and gangrenous; three or four openings into the urethra resulted, out of which he made his water. His scrotum had by this time regained its normal size. At last a little abscess occurred at the root of the penis, and was followed by urinary abscess. Then there was talk of removing the penis, and he came to this city. Here there were a number of opinions, but the feeling was that the trouble was lupous.

Then he came under my care. I thought he had a follicular sinus which came from behind a stricture, and on passing a bulbous sound, I found a stricture which warranted me in the conclusion that the urinary infiltration had got into the tissues and had resulted in the formation of a neoplasm in the scrotum. This slowly increased by almost imperceptible degrees, because of the gradual addition of urinary material until when the health became depressed, the tissues could resist no longer, but gave way at the most dependent point. The resulting inflammation closed up the sinus for an inch or two; then the urine settling at that point, gave rise to another abscess, each abscess closing up the line of the tract until the penis was reached. When he came to me, he had been suffering from a terrific amount of reflex irritation, keeping him from sleep, an amount of irritation which I

have never seen except in connection with stricture. His urethra had never received the slightest attention, and on passing a sound, during which I thought he would have had an epileptic convulsion, so sensitive was the urethra, I discovered three strictures, which I divided. The result was that he was relieved from the irritation which had been so disgusting. The relief was *immediate.* The next morning he said to me, "Doctor, for such a night of rest as I passed last night, I would be willing to undergo that operation every day." The abscess healed in ten days, and he became perfectly well.

I have told you this long story to show you how important it is to recognize and properly treat these hard, insensitive bunches. They may give the patient no trouble for the time being, but they will at some time or other. Whenever you find these bunches on the under side of the urethra, get into them as soon as you can. They are usually hard, insensitive, sometimes there are four or five little hard bunches. Sometimes these come from behind a stricture where you could pass a twenty-six or twenty-eight sound. Such a case I saw in consultation once. A young gentleman had one of these swellings in the urethra, and the attending surgeon cut down to the urethra, but not into it. Suddenly the swelling returned, and this time there was no mistake about it; a section was made into the urethra, a stricture divided and the gentleman made a good recovery. Here the follicular departure occurred where you could pass a twenty-six or twenty-seven sound.

Early operation is the rule in these cases. Remember that you are having urine leak into the tissues all the time through these little sinuses; it need not be large in quantity; it may be only enough to produce neoplastic material, but it goes on all the time and if the thing is not looked to and remedied by early operation, it will prove a source of trouble at some time or other, which trouble was clearly *preventable* by seasonable interference.

